# Molecular characterization of hepatitis B virus in Bangladesh reveals a highly recombinant population

**DOI:** 10.1371/journal.pone.0188944

**Published:** 2017-12-07

**Authors:** Saif Ullah Munshi, Thanh Thi Thanh Tran, Truc Nhu Thanh Vo, Shahina Tabassum, Nahida Sultana, Trang Hoa Nguyen, Munira Jahan, Chau Ngoc Le, Stephen Baker, Motiur Rahman

**Affiliations:** 1 Bangabandhu Sheikh Mujib Medical University, Shahbag, Dhaka, Bangladesh; 2 The Hospital for Tropical Diseases, Wellcome Trust Major Overseas Programme, Oxford University Clinical Research Unit, Ho Chi Minh City, Vietnam; 3 Centre for Tropical Medicine, Nuffield Department of Clinical Medicine, Oxford University, Oxford, United Kingdom; CEA, FRANCE

## Abstract

The natural history and treatment outcome of hepatitis B viruses (HBV) infection is largely dependent on genotype, subgenotype, and the presence or absence of virulence associated mutations. We have studied the prevalence of genotype and subgenotype as well as virulence and drug resistance associated mutations and prevalence of recombinant among HBV from Bangladesh. A prospective cross-sectional study was conducted among treatment naïve chronic HBV patients attending at Bangabandhu Sheikh Mujib Medical University, Dhaka, Bangladesh for HBV viral load assessment between June and August 2015. Systematical selected 50% of HBV DNA positive patients (every second patient) were enrolled. Biochemical and serological markers for HBV infection and whole genome sequencing (WGS) was performed on virus positive sample. Genotype, subgenotype, virulence, nucleos(t)ide analogue (NA) resistance (NAr) mutations, and the prevalence of recombinant isolates were determined. Among 114 HBV DNA positive patients, 57 were enrolled in the study and 53 HBV WGS were generated for downstream analysis. Overall, 38% (22/57) and 62% (35/57) of patients had acute and chronic HBV infections, respectively. The prevalence of genotypes A, C, and D was 18.9% (10/53), 45.3% (24/53), and 35.8% (19/53), respectively. Among genotype A, C and D isolates subgenotype A1 (90%; 9/10), C1 (87.5%; 21/24) and D2 (78.9%; 15/19) predominates. The acute infection, virulence associated mutations, and viral load was higher in the genotype D isolates. Evidence of recombination was identified in 22.6% (12/53) of the HBV isolates including 20.0% (2/10), and 16.7% (4/24) and 31.6% (6/19) of genotype A, C and D isolates, respectively. The prevalence of recombination was higher in chronic HVB patients (32.2%; 10/31 versus 9.1%; 2/22); p<0.05. NAr mutations were identified in 47.2% (25/53) of the isolates including 33.9% novel mutations (18/53). HBV genotype C and D predominated in this population in Bangladesh; a comparatively high prevalence of recombinant HBV are circulating in this setting.

## Introduction

There are an estimated two billion people with serological markers of present or past Hepatitis B virus (HBV) infection globally; 257 million of these are chronically infected[1]. The outcomes of acute HBV infection range from complete recovery to fulminant liver disease. A failure to clear HBV after acute infection may lead to either inactive or active chronic infection, which can induce hepatic insufficiency, end-stage liver disease including liver cirrhosis (LC) and hepatocellular carcinoma (HCC) [2, 3].

Current classification segregates HBV into 10 different genotypes (A-J; segregated by <7.5% genomic sequence diversity); these are further classified into 40 different subgenotypes, which are separated by >4% genomic sequence diversity [4]. The dominant HBV genotypes and subgenotypes differ by geographical location, transmission dynamics, disease progression, and response to antiviral therapy [5]. The clinical progression of HBV infection is dependent on multiple factors, which includes age when infected, genetic factors, and the infecting genotypes and subgenotypes [6]. Notably, factors such as genotype and subgenotype as well as HBV e antigen (HBeAg) status, viral load, drug resistance mutations in the reverse transcriptase (RT) domain of polymerase gene, and mutations in the basal core promoter (BCP) precore (PC) and core gene have a major influence in determining disease progression and treatment outcome [7–9]. End-stage liver disease, and poor response to interferon therapy is commonly observed in chronic HBV infection associated with genotypes C and D [5]. It has also been observed that horizontal HBV transmission is more common with genotypes A and D [5]. Further, a number of genetic characteristics including mutations in *preS*1, *preS*2, and *S* genes have been shown to be associated with viral replication, progression of liver disease including HCC, occult HBV infections (OBI), immune escape, and therapy escape [10–13]. Mutations in the core promoter (CP) have been associated with severity of liver disease; a G1896A mutation in the precore and core gene creates a premature stop codon at position 28 and abolishes the synthesis of HBeAg [10, 14].

Bangladesh is a country with intermediate endemic HBV and a chronic HBV carriage rate of 2–6%. The prevalence of chronic HBV among the general population and various high-risks groups, including intravenous drug users, ranges from 0.8% to 6.2% [15] [16]. However, data regarding the prevalence of HBV genotypes and subgenotypes, the prevalence of recombinant viruses, virulence-associated characteristics such as drug resistance mutations are limited from Bangladesh. In order to address this paucity of data, we performed a prospective cross sectional study to determine the dominant HBV genotypes and subgenotypes. We additionally assessed the prevalence of recombination, resistance associated mutations, and the prevalence of virulence mutations in HBV in this setting.

## Materials methods

### Study population

A prospective cross-sectional study was conducted among treatment naïve patients attending the Bangabandhu Sheikh Mujib Medical University (BSMMU), a tertiary care hospital in Dhaka, for HBV viral load testing between June and August 2015. All patients attending at BSMMU for HBV viral load assay and providing written informed consent were eligible for enrollment in the study. Parental or guardian consent were collected for patient <18 years of age. Systematically selected 50% (every second) of the HBV DNA positive patients was enrolled in the present study. Venous blood samples were collected from enrolled patients for biochemical, virological, and molecular testing. Plasma samples were stored at -86°C before being shipped to Oxford University Clinical Research Unit, Ho Chi Minh City, Vietnam for further analysis. Clinical chemistry results of patients were obtained from the hospital database for analysis. The study was approved by Bangabandhu Sheikh Mujib Medical University ethical review committee (Approval No. BSMMU/2014/10612).

### HBV, HCV, HIV serology

All plasma samples were tested for HBs Ag, HBe Ag, anti HBs, anti HBe, anti HBcTotal, and anti HBc IgM using serological tests as per the manufacturer’s recommendation (Beijing Wantai Biological Pharmacy Enterprise Co., Ltd., Beijing, China). Serum samples were classified as being from acute HBV infections (HBs Ag positive, anti HBcTotal positive, anti HBC IgM positive, anti HBs negative), or chronic HBV infections (HBs Ag positive, anti HBcTotal positive, and anti HBc IgM negative and anti HBs negative) according to the CDC guidelines for the interpretation of hepatitis B serological test results (http://www.cdc.gov/hepatitis/HBV/PDFs/SerologicChartv8.pdf). All samples are screened for anti hepatitis C (HCV) antibody by ELISA (Beijing Wantai Biological Pharmacy Enterprise Co., Ltd., Beijing, China) and for anti human immunodeficiency virus (HIV) antibody by ELISA (DIALAB ELISA, Biorad, France) as per manufacturer’s recommendation.

### HBV DNA sequencing

Viral load was determined by real-time PCR using the Single Step HBV DNA assay kit (Genebio Inc. Ltd. USA) as per the manufacturer’s instructions. Viral DNA was extracted from 200μL of plasma using QIAamp viral DNA extraction kit (QIAgen GmbH, Hilden, Germany) before elution in 50μL tris borate EDTA (TBE) buffer. HBV nucleic acid was prepared for genome sequencing by PCR amplification of four overlapping fragments (800bp to 1.2 kb) using P1-P2, P3-P4, P5-P6, and P7-P8 primers (P1: 5'-TTTTTCACCTCTGCCTAATCA-3'; P2: 5'-TTGGGATTGAAGTCCCAA TCTGG-3'; P3: 5'-GGGTCACCTTATTCTTGG-3'; P4: 5'-ATAACTGAAAGCCAAACAGTG GG-3'; P5: 5'-GTCTTCTTGGTTGTTCTTCTAC-3'; P6: 5'-GCAGCACAGCCTAGCAGCCAT GG-3'; P7: 5'-CCATACTGCGGAACTCCTAGC-3'; P8: 5'-CAATGCTCAGGAGACTCTAAG GC-3') [17]. The PCR amplification reactions were performed in 40μL volumes containing 50mM Tris-HCl (pH 8.3), 50mM KCl, 1.5mM MgCl2, 200mM deoxynucleoside triphosphates (dNTPs), 1U of Taq DNA-Pwo Polymerase (Expand High Fidelity assay, Boehringer Mannheim) and 30pmol of primer. Amplification was performed for 35 cycles at 94°C for 1 minute, 58°C for 1 minute and 72°C for 1 minute in a thermal cycler (ABI 9800). PCR amplicons were visualized by 1% agarose electrophoresis and stained with Nancy 520 DNA gel stain.

PCR amplicons were purified using the QIAamp PCR product purification kit (QIAgen GmbH, Hilden, Germany). The eluted DNA was quantified by a fluorescence-based dsDNA quantification method using the Quant-iT dsDNA Assay Kit in a Qubit fluorometer (Invitrogen). For sequencing, genomic fragments were pooled into equal quantities of each individual PCR amplicon. One nanogram of pooled DNA from each individual sample was subjected to library preparation using the Nextera XT DNA sample preparation kit (Illumina, San Diego, CA, USA), in which each sample was assigned to a unique barcode sequence using the Nex-tera XT Index Kit (Illumina). Sequencing of the libraries was performed using MiSeq reagent kit v2 (300 cycles, Illumina) on an Illumina MiSeq platform. All samples were sequenced in a single run. The Illumina fastq sequence files were assembled using Genious 8.0.5 software package (Biomatters Ltd, Auckland, New Zealand) utilizing a reference-based mapping tool after primer sequences clipping (i.e. the consensus sequence was obtained by mapping individual reads of each sample to a reference sequence). A minimum variant frequency of 5% and 500-fold coverage were chosen as cut-off values and all analysis was done on dominant/consensus variants. The resulting sequences were deposited in GenBank under accession numbers MF925358 to MF925410.

### HBV recombination and phylogenetic analysis

One hundred and three HBV whole genome sequences (WGS) representing all 10 genotypes and at least two sequences for each of the subgenotypes were downloaded from Genbank and combined with 53 HBV WGS from the current study. These 156 sequences were subjected to recombination and phylogenetic analysis (S1 File) [4, 18]. All sequences were analyzed for possible recombination by RDP4 v 4.55 software. Any recombination detected by at least 5 of the 7 programs (RDP, Geneconv, Bootscan, Maxchi, Chimaera, Siscan, and Topol) was considered as true recombination. RDP4 v4.55 standard default setting was used except for Bootscan and Siscan the window sizes 300bp, step size 30 were used. Data on the type of recombination, recombination breakpoint (start and end point), homology with major parent & minor recombinant parent, the size of the recombinant fragment, and the location of the recombination were determined.

For phylogenetic analysis, all 156 complete genome sequence were aligned using MUSCLE in the Genious software package (S1 File) [19]. Phylogenetic analysis was conducted in two stages. In the first stage, analysis was conducted using the full length genome sequences (data not shown). As recombination analysis revealed a highly recombinant population and the limits of the regions susceptible to recombination, we conducted second stage of analysis to determine the evolutionary relationships between the isolates. In this stage, two partial sequence alignments were created; the first alignment contained WGS without the recombination susceptible region (1–1,272bp and 2,028–3,215bp) and the second set of alignment contained the recombination susceptible region only (1,273bp—2,027 bp). The sequence alignments were subjected to Jmodel test to identify the best model for phylogenetic analysis. The suggested nucleotide substitution model (GTR+G+I) was subsequently used in the phylogenetic analysis using RAxML v7.2.8 (available in Genious package). To confirm the reliability of phylogenetic tree, bootstrap resampling and reconstruction were performed 1000 times [20].

### HBV mutation analysis

The HBV polymerase gene (nt 2306..1623) consists of four domains (terminal protein (TP) domain; nt 2307–2843, spacer; 2844..3215, 1..135, reverse transcriptase (RT)domain; nt 136..1167, and RNaseH (RH) domain; nt 1168..1623. The RT domain (136bp to 1167bp) was analyzed for the presence of 42 potential nucleos(t)ide analogue (NA) resistance (NAr) mutations. This included the primary drug resistance mutation (rt169, rt180, rt181, rt184, rt194, rt202, rt204, rt236, and rt250), the secondary drug resistance mutations (rt80 and rt173), the putative NAr mutations (rt53, rt54, rt82, rt84,rt85, rt91, rt126, rt128, rt139, rt153,rt166, rt191,rt200, rt207,rt213, rt214,rt215,rt217, rt218, rt221, rt229, rt233, rt237, rt238, rt245, and rt256), and the pretreatment mutations (rt38, rt124, rt134, rt139, rt224, and rt242) as previously described [21]. The distribution of mutations in six functional regions (F, A, B, C, D, and E) of RT domain and the five the regions (F-A, A-B, B-C, C-D, and D-E) connecting them were analyzed. The RT domain was also analyzed for genotype dependent AA polymorphism at rt38, rt53, rt54, rt91, rt124, rt126, rt139, rt153, rt221, rt224, and rt256 positions [21].

The *preS2/S1* regions were analyzed for *preS1* deletion, *preS1* mutations (A2962G, C3026A/T, C2964A, and C3116T), *preS2* start codon deletion, and preS2 mutations (T53C). The *S* gene was analyzed for mutations in major hydrophilic region (MHR) (99aa-169aa) including the “a” determinant region (Y/L100C/I, Q101H/R, T113S, T115N, I/T126S/N, T127P, A128V, A143L, G145R, R160K, Y161F, E164D, and A168V), and other mutations associated with increased risk of HCC (N3S, R24K, P56Q, P62L, F85C, L126T/S, A168V, V184A, and S204R) [22–25] [26] [27]. Mutations in BCP (C1653T, T1753C, G1757A, A1762T, G1764A, C1766G, and T1768A), and the PC/core region associated with HCC (G1896A, G1899A, A2159G and A2189C/T, and G2203A/T) were also analyzed.

All data (socio demographic, biochemical, and virological) was recorded and analyzed using the Statistical Package for the Social Sciences (IBM SPSS version 23, NY, USA). Pearson’s Chi squared test was used for the comparison qualitative variables and Mann—Whitney U test for ordinal scale variables. The one-way ANOVA test was used for comparing the significance between three or more groups. A *p* value <0.05 was inferred to indicate statistical significance.

## Results

From June to August 2015, a total of 274 treatment naive patients attending BSMMU for HBV viral load measurement were invited to participate the study. Among these, 159 patients provide informed consent to join the study. Of these 159 patients, 114 were HBV DNA positive and 57 patients (50%; every second patient) were enrolled in this study. The sex, demographics, liver enzyme profiles, HBV serology, and hepatitis status of all 57 patients are presented in Table 1. Approximately 80% (47/57) of the patients were male and the median age was 32.2 years (12 to 65 years; IQR 20); 38.5% (22/57) of patients had an acute HBV infection and 61.5% (35/57) had a chronic HBV infection. AST, ALT, and serum bilirubin was significantly higher in those with an acute HBV infection (*p*<0.05). HBe Ag seropositivity was higher amongst the acute HBV patients than the chronic HBV patients, although this was not statistically significant. The median viral loads of the acute HBV infection cases were significantly higher than chronic HBV infection (2.9x10^6^ versus 3.2x 10^3^; *p*<0.001) (Table 1). All patients were HCV and HIV negative (data not shown).

**Table 1:** Socio demographic, biochemical, serological and virological profile of 57 treatment naïve acute and chronic HBV DNA positive patients enrolled in the study during June to August 2014.

Variable		All	Acute infection	Chronic infection	*p* value
		% (n)	% (n)	% (n)	
		N = 57	38.5 (22)	61.5 (35)	
Age ^a^		32.25 (12–65)	29.5 (19–65)	28.0 (12–60)	0.104 ^c^
Sex					
	Male	82.5 (47)	90.9 (20)	77.1 (27)	
	Female	17.5 (10)	9.1 (2)	22.9 (8)	
Biochemical (mean; min–max)					
	AST(SGOT) ^a^	74.82 (18.0–482.0)	101.95 (22.0–482.0)	57.2 (18.0–319.0)	0.01^b^
	ALT (GSPT) ^a^	87.69 (22.0–405.0)	116.27 (28.0–405.0)	69.2 (22.0–313.0)	0.002 ^b^
	Creatinin ^a^	73.2 (34.0–134.0)	75.8 (48.0–134.0)	71.4 (34.0–119.0)	0.486 ^b^
	Bilirubin ^a^	21.4 (2.5–189.1)	36.2 (2.9–189.1)	5.7 (2.5–21.0)	0.006 ^b^ *
HBV Serology					
	Anti HBs	5.3 (3)	4.5 (1)	5.7 (2)	0.847 ^c^
	HBe Ag	33.3 (19)	50.0 (11)	22.9 (8)	0.034 ^c^ *
	Anti HBe	47.4 (27)	36.4 (8)	54.3 (19)	0.187 ^c^
	Anti HBc Total	96.5 (55)	95.5 (21)	97.1 (34)	0.736 ^c^
	Anti HBc IgM	38.6 (22)	100 (22)	0.0 (0)	0.001 ^c^ **
Genotype (n = 53)					
	A	18.9 (10)	13.6 (3)	22.6 (7)	0.422 ^c^
	C	45.3 (24)	45.5 (10)	45.2 (14)	0.984 ^c^
	D	35.8 (19)	40.9 (9)	32.3 (10)	0.527 ^c^
HBV viral load^c^		7.3x10^3^ (1.1x10^2^–4.9x10^8^)	2.9x10^6^ (3.9x10^2^–4.9x10^8^)	3.2x 10^3^ (1.1x102–5.7x107)	0.001 ^b^**

^a^ = mean (minimum–maximum)

^b^ = Mann-Whitney U test

^c^ = Person’s chi square test

* = <0.05

** = <0.01

**Fig 1:**
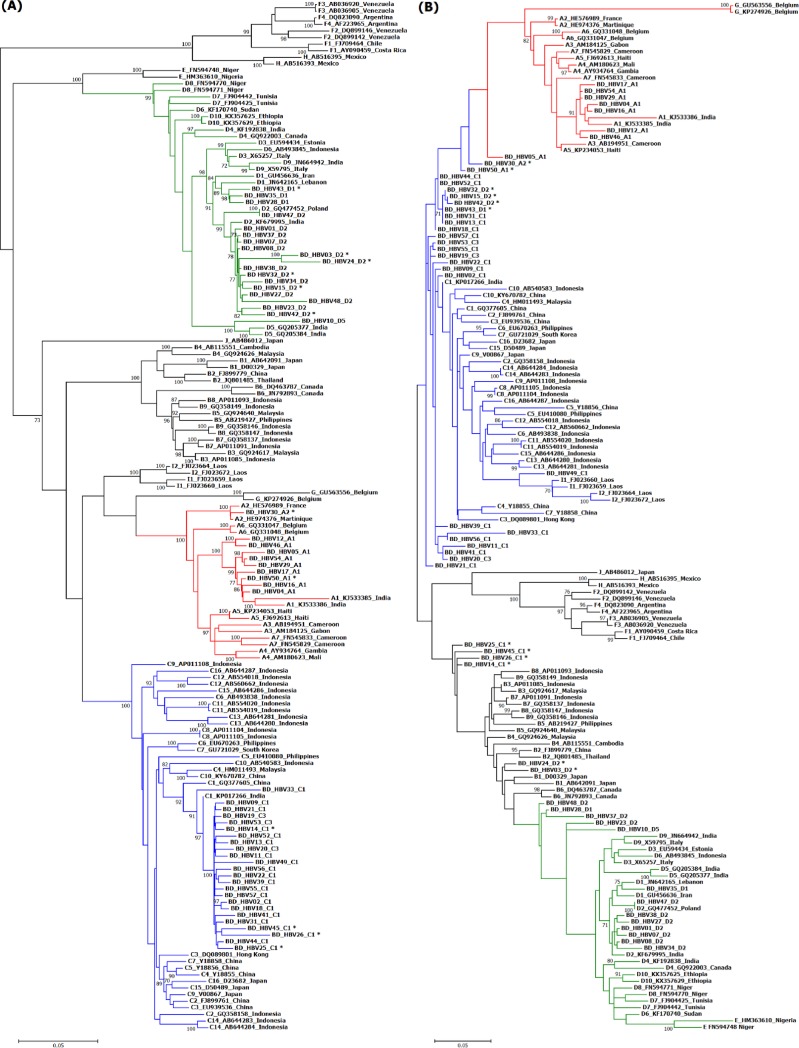
A midpoint rooted tree showing the relationship between the Bangladeshi HBV genome sequences with 103 reference sequences. The tree was constructed using RAxML v7.2.8 available in Geneious software using GTR+G+I nucleotide substitution model with 1000 bootstrapping replicates. Bootstrap values greater than 70% are shown at the branch nodes. The Bangladeshi HBV strains are presented as BD HBV followed by isolate number, genotype and subgenotype and reference genomes are presented as genotype, subgenotype followed by Gene Bank accession number and country of origin. The scale bar indicates the number of nucleotide substitution. (A) presents the full-length genome excluding the recombination susceptible region (1–1272 and 2028–3215 bp). Isolates with recombination are marked with a asterics and are clustered with major parent genotype. (B) present the recombination susceptible region (1273 to 2027 bp). Isolates with recombination are marked with a asterics and are clustered with minor parent genotype.

HBV DNA positive samples (n = 57) were subjected to WGS, resulting in 53 HBV whole genome sequences. The mean length of the genome sequences was 3,202 bp (range: 3,125–3,227bp; Genotype A; 3,221–3,221bp, Genotype C; 3,125–3,227bp, and genotype D; 3,131–3,182bp). The genotypes and subgenotypes of all isolates as determined by phylogenetic analysis are shown in Fig 1A. Among the 53 HBV whole genome sequences, 18.6% (10/53) were genotype A, 42.1% (24/53) were genotype C, and 33.3% (19/53) were genotype D. Of the 10 genotype A HBV, 90% (9/10) were subgenotype A1, and 10% (1/10) were subgenotype A2. Of the 24 genotype C viruses, 87.5% (21/24) were subgenotype C1 and 12.5% (3/24) were subgenotype C3. Of the 19 genotype D viruses, 15.7% (3/19) were subgenotype D1, 78.9% (15/19) were subgenotype D2, and 5.2% (1/19) were genotype D5 (Fig 1A). Besides this, HBV isolates from Bangladesh clustered with isolates from neighboring India.

**Fig 2:**
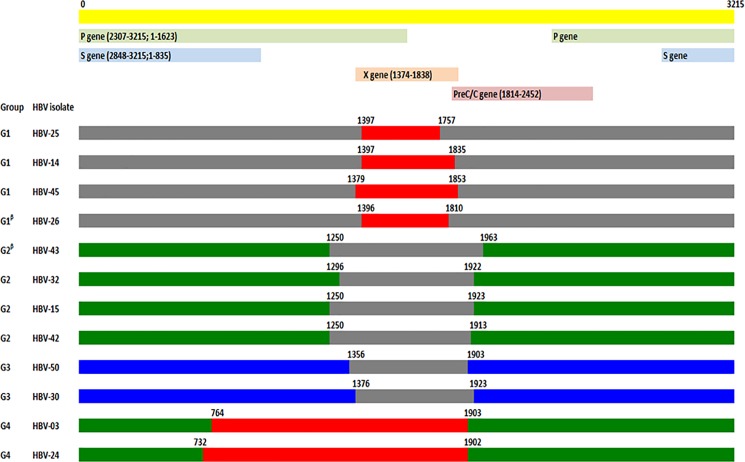
Schematic representation of the mosaic structure of the HBV recombinant sequences of the present study. The recombinant sequences are divided into four groups (G1 to G4); based on the major and minor parent of recombination fragments. The recombinant between C/B, D/C, A/C, D/B genotypes were presented as Group1, 2, 3, and 4 respectively. Genotype A sequence is presented in blue, genotype B in red, genotype C in gray and genotype D in green. A linear physical map of the HBV genome including the position of different gene(s) is depicted. Numbering starts from the hypothetical EcoRI restriction site.

Recombination analysis using RDP identified evidence of recombination in 22.6% (12/53) of the HBV isolates including 20.0% (2/10), and 16.7% (4/24) and 31.6% (6/19) of genotype A, C and D isolates, respectively. The recombination events were classified into four groups based on the size of the recombinant fragment and the major parent genotypes; in group 1 (4 isolates), group 2 (4 isolates), group 3 (2 isolates), and group 4 (2 isolates) recombination was evident between genotype C/B, D/C, A/C and D/B, respectively. The recombinant groups, types, fragment lengths, breakpoints, major and minor parents, and sequence homologies with these parents are shown in Fig 2, Fig 1B and S1 Table. The majority of the recombination events were identified in the X gene and in the early part of the PC/C gene. The number of recombination events was significantly greater in the HBV associated with chronic infections (32.2% (10/31) versus 9.1% (2/22); *p*<0.05).

We identified an 18bp (incorporating the start codon) *preS1* deletion in 39.6% (21/53) of HBV samples, which included all the genotype D isolates (*p*<0.001). A *preS2* M1V/T start codon mutation was identified in two isolates. The presence of an F22L mutation in *preS2* was significantly more common in genotype A viruses than other viruses (50% (5/10); *p*<0.002) (Table 2). Mutations in *S* gene can be found within or outside the MHR region. Mutations in N3S, L126T/S, A168V were significantly more common in genotype C HBV (*p*<0.001) and genotype D HBV (*p*<0.001) than other genotypes (Table 2). MHR mutations were identified in 17.5% (10/53) isolates including 10.5% (6/53) associated with HBsAg detection failure, 12.7% (7/53) associated with escape mutants, 7% (4/53) associated with therapy failure, and 17.5% (10/53) associated with OBI. However, there was no significant difference in the distribution of MHR mutations between genotypes (Table 3).

**Table 2:** Mutations in the preS2/S1/S gene reported to be associated with HCC.

Variable			All	Genotype A	Genotype C	Genotype D	p value ^d^	Reference
			% (no)	% (no)	% (no)	% (no)		
			N = 53	n = 10	n = 24	n = 19		
Gene	Nucleotide	Amino acid						
Genome length			3125–3227	3221–3221	3125–3227	3131–3182		
PreS1 gene (nts 2848–3204)								
	Pre S1 deletion		39.6 (21)	0 (0)	8.3 (2)	100 (19)	0.000**	[13] [12]
	6 bp insertion in 2374		20.8 (11)	90 (9)	8.3 (2)	0 (0)	0.000**	
PreS2 gene (nts 3205–154)								
	Start codon	M1V/T/I	5.7 (3)	20 (2)	4.2 (1)	0 (0)	0.010*	[13] [12]
	PreS1/S2 continuous		3.8 (2)	20 (2)	0.0 (0)	0 (0)	0.010*	[13]
	T53C	F22L	15.1 (8)	50 (5)	12.5 (3)	0 (0)	0.002*	[13] [12]
S gene mutation associated with HCC (nts 155–835)								
	A162G	N3S	35.8 (19)	0 (0)	75.0 (18)	5.3 (1)	0.000**	[12]
	G225A	R24K	5.7 (3)	0 (0)	0 (0)	15.7 (3)	0.058	[28]
	C321A	P56Q	1.9 (1)	0 (0)	0 (0)	5.3 (1)	0.417	[28]
	C339T	P62L	3.8 (2)	0 (0)	8.3 (2)	0 (0)	0.297	[29]
	T408G	F85C	1.9 (1)	0 (0)	0 (0)	5.3 (1)	0.417	[30]
	T531C/G	L126T/S	66.0 (35)	100 (10)	29.1 (7)	94.7 (18)	0.000**	[12]
	T657C	A168V	33.9 (18)	0 (0)	0 (0)	94.7 (18)	0.000**	[27]
	A706C	V184A	0 (0)	0 (0)	0 (0)	0 (0)	0.116	[12]
	T766A	S204R	1.9 (1)	0 (0)	0 (0)	5.3 (1)	0.162	[12]
	Stop codon on S	3.8 (2)	10 (1)	4.2 (1)	0 (0)	0.417		

^d^ = One way ANOVA test

* = <0.05

** = <0.001

**Table 3:** Mutations in MHR (aa 99–169) including “a” determinant region (aa 124–147).

Amino acid position	All	Genotype A	Genotype C	Genotype D	Reference
	% (no)	% (no)	% (no)	% (no)	
	N = 53	n = 10	n = 24	n = 19	
Y100C^IV^	3.8 (2)	0 (0)	8.3 (2)	0 (0)	[22]
Q101K	3.8 (2)	0 (0)	8.3 (2)	0 (0)	[22]
Q101R ^IV^	1.9 (1)	0 (0)	4.2 (1)	0 (0)	[22]
L100I ^IV^	1.9 (1)	0 (0)	4.2 (1)	0 (0)	[22]
T113S ^IV^	1.9 (1)	0 (0)	4.2 (1)	0 (0)	[22]
T115N ^II^^,^^IV^	1.9 (1)	0 (0)	0 (0)	5.3 (1)	[22]
**I/T126N** ^**I**^^,^ ^**II**^^,^^**III**^^,^^**IV**^	**3.8 (2)**	**0 (0)**	**4.2 (1)**	**5.3 (1)**	[22][23]
**I/T126S** ^**I**^^,^ ^**II**^^,^ ^**IV**^	**1.9 (1)**	**0 (0)**	**4.2 (1)**	**0 (0)**	[22][23]
**T127P**	**5.7 (3)**	**0 (0)**	**0 (0)**	**15.8 (3)**	[22]
**A128V**	**7.6 (4)**	**0 (0)**	**0 (0)**	**21.0 (4)**	[22]
**S143L** ^**I**^^,^ ^**II**^^,^ ^**IV**^	**1.9 (1)**	**0 (0)**	**0 (0)**	**5.3 (1)**	[22][24][26]
**G145R** ^**I**^^,^ ^**II**^^,^ ^**III**^^,^ ^**IV**^	**3.8 (2)**	**0 (0)**	**8.3 (2)**	**0 (0)**	[22][24][26]
R160N	1.9 (1)	0 (0)	4.2 (1)	0 (0)	[22]
Y161F	1.9 (1)	10 (1)	0 (0)	0 (0)	[26]
E164D	1.9 (1)	0 (0)	4.2 (1)	0 (0)	[26]
A168V^IV^	1.9 (1)	0 (0)	0 (0)	5.3 (1)	[27]

I; Mutations associated with HBsAg detection failure

II; mutations associated with escape mutant

III; mutations associated with therapy escape

IV; mutations associated OBI. Mutation in “a” determinant region is marked in bold. Amino acid positions are relative to HBV reference sequence

GenBank accession number AB014381

BCP mutations at C1653T T1753C, G1757A, A1762T, G1764T and C1766G were investigated and 70.2% (40/53) of all isolates, which included 100% (10/10), 66.6% (16/24), and 73.6% (14/19) of genotypes A, C, and D viruses, respectively. There was no significant difference in the combination of BCP mutations among different genotypes (Table 4). A G1896A mutation in the PC gene was present in 26.4% (14/53) of viruses, which included 47.4% (9/19) of the genotype D viruses and none of the genotype A viruses and 20.8% (5/24) of genotype C viruses.

**Table 4:** Mutations in the BCP, PC/core gene associated with HCC in 53 HBV isolates from Bangladesh.

Variable		All	Genotype A	Genotype C	Genotype D	*p* value ^d^
		% (no)	% (no)	% (no)	% (no)	
		N = 53	n = 10	n = 24	n = 19	
	Nucleotide					
Mutation in BCP region						
	C1653T	5.7 (3)	10 (1)	8.3 92)	0 (0)	0.297
	T1753C	13.2 (7)	20 (2)	16.7 (4)	5.3 (1)	0.443
	G1757A	37.7 (20)	30 (3)	29.2 (7)	52.6 (10)	0.258
	A1762T	32.1 (17)	60 (6)	29.2 (7)	21.1 (4)	0.097
	G1764A	37.7 (20)	70 (7)	33.3 (8)	26.3 (5)	0.059
	A1762T/G1764A	32.1 (17)	60 (6)	29.2 (7)	21.1 (4)	0.097
	C1766G	1.9 (1)	0 (0)	00.0 (0)	5.3 (1)	0.417
	T1768A					
Mutation in PC/Core						
	G1896A	26.4 (14)	0 (0)	20.8 (5)	47.4 (9)	0.014*
	G1899A	8.8 (5)	0 (0)	8.3 (2)	15.8 (3)	0.387
	A2159G	5.7 (3)	0 (0)	12.5 (3)	0 (0)	0.152
	A2189C/T	50.1(27)	0 (0)	33.3 (8)	100 (19)	0.000**

^d^ = One way ANOVA test

* = <0.05

** = <0.01

Reverse transcriptase sequence analysis identified genotype dependant AA polymorphisms, previously reported NAr mutations, and additional novel NAr mutations. The genotype dependant AA polymorphism was identified in 11 sites (Table 5). The presence of threonine at rt38, serine at rt 53, tyrosine at rt 124, isoleucine at rt 224 were significantly associated with genotype C (*p*<0.0001). Similarly, the presence of isoleucine at rt 91, glutamine at rt 139, tryptophan at rt 153, tyrosine at rt 221 and aspartic acid at rt 53, histidine at rt 54, arginine at rt 126, and cysteine at rt 256 were all significantly associated with genotype A and genotype D viruses respectively (all *p*< 0.0001) (Table 5).

**Table 5:** Genotype–dependent AA polymorphic sites in RT domain of HBV polymerase gene identified in this study.

		rt38			rt53						rt54			rt91		rt124			rt126			rt139				rt153			rt221		rt224		rt256	
		A^b^	E^a^	T	D	I	K^a^	N^a^	S	V^a^	H	T	Y^a^	I	L^c^	H^b^	N	Y	H	R	Y^a^	D^a^	H^a^	N	Q	Q^a^	R	W	F	Y^c^	I	V^b^	C	S
**Genotype A**	N = 10	10	0	0	0	5	0	0	1	4	0	10	0	10	0	1	9	0	7	0	3	0	0	0	10	0	0	10	0	10	0	10	0	10
**Genotype C**	N = 24	1	0	23	0	0	0	0	24	0	0	24	0	2	22	2	0	22	24	0	0	1	0	23	0	2	21	1	24	0	22	2	0	24
**Genotype D**	N = 19	16	1	2	14	0	1	3	1	0	14	2	3	1	18	18	1	0	4	15	0	0	1	18	0	0	19	0	17	2	0	19	17	2
	P value	p < 0.0001			p < 0.0001						p < 0.0001			p < 0.0001		p < 0.0001			p < 0.0001			p < 0.0001				p < 0.0001			p < 0.0001				p < 0.0001	

A: alanine; E: glutamic acid; T: threonine; D: aspartic acid; I: isoleucine; K: Lysine; N: asparagine; S: serine, H: histidine; Y: tyrosine; R: Arginine; Q: Glutamine; W: Tryptophan; F: Phenylalanine; G: glycine; F: phenylalanine; V: valine; C: cystine.

^a^ Described as naturally occurring polymorphic mutations in this study

^b^ Pretreatment mutation found to be Genotype–dependent AA

^c^ Putative NAr mutation found to be Genotype–dependent AA

The NAr mutation was identified in 47.2% (25/53) of isolates, including previously reported mutations in 22.6% (12/53) of viruses and novel NAr mutations in 33.9% (18/53) of the isolates. 28.3% (15/53) of the viruses had at least one NAr mutations and 17.5% (10/53) had two or more mutations. The prevalence of previously reported primary, secondary, putative and pretreatment mutations, were 1.9% (1/35), 3.8% (2/53), 15.1% (8/53), and 7.6% (4/53), respectively (S2 File). The prevalence of any previously reported mutations were significantly higher in the genotype D viruses than the other genotypes (Genotype A; 10% (1/10), Genotype C; 20.8% (5/24), and Genotype D; 31.5% (6/19) (*p*< = 0.05).

NAr mutations were further characterized for their presence in RT domains or interdomian regions. Interdomian mutations were more common than domain mutations for previously reported (74.6%; (11/15) versus 26.4%; (4/15) and novel mutations (82.7% (19/23) versus 17.3% (4/23) (S1 Fig).

## Discussion

A description of genotypes and subgenotypes is important for a better understanding of the epidemiology, transmission, virulence potential, and clinical outcome of HBV infections [5]. One of the key criteria for assigning a virus to a subgenotype is generating a whole genome sequence. Here, we analyzed a collection of HBV genome sequences collected in a single healthcare facility in Bangladesh. To our knowledge, this is the first study reporting genotypes and subgenotype of HBV in Bangladesh through WGS. Considering the prevalence of chronic HBV and the limited availability facilities for characterizing HBV in Bangladesh, this genotyping and subgenotype data from the Bangladeshi population is important for clinical management decision making, disease modeling, and health resource allocation for the management of chronic HBV [31].

The majority of the HBV in our study belonged to genotype C and D, which have a higher risk of HCC and chronic infection then genotype A and B. The progression to chronic HBV infection has been shown to be commonly associated with genotypes A and D then with other genotypes [31]. Our data is in agreement with recently published HBV genotyping data from Bangladesh, and data from neighboring countries including India [32] [33]. Genotypes A and D are known to be horizontally transmitted, more than half of the HBV identified here were genotype A or D, indicating possible horizontal transmission through blood or blood products [31]. Additionally, we observed a high degree of recombination among these HBV isolates; it is not apparent if this recombination occurred a result of co-infection/super infection with two genotypes within the patient or if the patient was infected with the recombinant strain. In half of the recombinant sequences, the recombination fragment (minor parent) was a genotype C virus. As the prevalence of genotype C HBV is high in Bangladesh, it is possible that patients were co-infected/super infected with two genotypes. The majority of the recombination breakpoints identified here, were in agreement with previous studies where recombination breakpoint hotspots have been observed in the X gene and the preCore/core gene [34]. Although the prevalence of recombination (HBV B/C) in HBV genotype B/Ba (B2-B5) from Vietnam, China, Hong Kong, Indonesia, and Thailand is high [35], the prevalence of genotype D/C or D/B recombination are relatively infrequent [34].

We chose to select patients who were treatment naïve to identify preexisting drug resistance mutations not likely influenced by treatment selective pressure. It is not unexpected that the majority of the viruses did not harbor primary or secondary drug resistance associated mutations, as the majority of the patients were treatment naive. Approximately half of the HBV had putative and pretreatment NAr mutations, including a third of the viruses exhibiting a novel mutation, and the prevalence of novel mutations was higher in the genotype D viruses. Eleven genotype-dependent AA polymorphic positions were identified for A-, C- and D- genotypes; similar observations have been reported previously. The cause of novel amino acid substitution associated with NAr and the aa dependant polymorphism is not known, however, it has been suggested that such mutations may be associated with the evolution and adaption of HBV in a defined population [21]. We identified an isoleucine at rt91 and tyrosine at rt221 is genotype A dependant; however, these positions (isoleucine at rt91 and tyrosine at rt22) have been reported as putative NAr mutations. Therefore, potential NAr mutations need further investigation regarding nucleos(t)ide resistance *in vitro* and *in vivo*. Moreover, the AA sites in interdomians displayed the highest mutation frequency (S1 Fig). It is likely that the interdomians are less crucial for RT function and antiviral resistance, rather the mutations within which might be driven by host immune responses [36].

All genotype D isolates and 8% of the genotype C isolates had an 18bp *preS1* deletion. Longitudinal studies have shown that the *preS* deletion mutations occur during the long course of liver disease, but not at the beginning of HBV infection [12]. It is thought that such deletions evolve during the course of long lasting infections and are associated with higher risk of HCC. We found that HCC associated mutations in at A168V and V184A were significantly higher in genotype D isolates [12]. Analysis of MRH region showed that 17% of the isolates had mutations in the “a” determinant region. These mutations can affect the antigenicity of HBsAg, and have shown to be responsible for false-negative results by commercial assays for HBsAg, evasion of anti-HBV immunoglobulin therapy, and evasion of vaccine induced immunity. These “vaccine-escape” mutants are more common in countries with high rates of endemic infections and universal immunization programs [23].

Mutations in BCP region, specifically the G1762A/G1764A double mutation, have suggested to be closely associated with HCC [13]. One-third of the isolates in the present study across all genotypes harbored these mutations, indicating the presence of HBV with increased risk for HCC in Bangladesh. The G1896A mutation in the precore/core gene results in a stop codon in 28 aa of core gene, and has been shown to be associated with fulminant hepatitis [10]. Approximately half of the genotype D isolates in this study harbored this mutation, indicating a potential for hepatic flare in these patients.

This study has limitations. First, specimens was collected over a short period of time and from a single tertiary care hospital and may not be representative of the population in Dhaka or Bangladesh as a whole. Second, a lack of data on the clinical presentation from the patients whom the sample was collected limits the clinical relevance of the viral subgenotype. Longitudinal studies on patients with specific subgenotype infection are essential to fill this knowledge gap.

## Supporting information

S1 File The HBV reference genome sequence and sequences isolated from Bangladesh in this study used for phylogentic analysis. Genotype, subgenotype, GenBank accession number and country of origin of the reference sequences used for phylogenetic analysis. BD_HBV followed by isolate number, GenBank accession number and subgenotype of the HBV isolates from this study.
(DOCX)

S2 File Potential NAr mutations including reported NAr mutations and novel NAr mutations identified in the present study. Potential NAr mutations including reported NAr mutations and novel NAr mutations identified in the present study among different genotypes are presented.
(DOCX)

S1 Table Recombination analysis of the 12 HBV isolates from the study using RDP4 v 4.55 program. HBV isolates were considered recombinant if detected by 5 out of 6 program (RDP, BootScan, Max Chi, Chimaera, SisScan and Topol). Recombinant isolate, recombination group major and minor parents and identity, recombinant break points, size of the recombinant fragment and location of the recombination are presented.
(DOCX)

S1 Fig Distribution of all NAR mutations identified in RT region. Schematic presentation of six HVB RT domain functional regions and (F, A, B, C, D, and E) five regions (F-A, A-B, B-C, C-D, and D-E) connecting the functional regions. Functional regions are presented as box and regions between functional regions as lines. The start and end amino acid positions of each functional region are presented at the top of each box. Novel mutations are presented as brown bars and previously reported mutations as blue bars.
(DOCX)
