# Supplementary material for: Molecular characterization of hepatitis B virus in Bangladesh reveals a highly recombinant population
Source: PLoS One. 2017 Dec 7;12(12):e0188944. doi: 10.1371/journal.pone.0188944 (PMC5720799; doi:10.1371/journal.pone.0188944)
Supplement: S1 File — Genotype, subgenotype, GenBank accession number and country of origin of the reference sequences used for phylogenetic analysis. BD_HBV followed by isolate number, GenBank accession number and subgenotype of the HBV isolates from this study. (DOCX) [file pone.0188944.s001.docx]

S1 File: List of reference and HBV isolates from the present study

The 103 HBV reference genome sequence including all genotype and at least two sequence for each subgenotype used for phylogenetic analysis: A1_KJ533385_India; A1_KJ533386_India; A2_HE576989_France; A2_HE974376_Martinique; A3_AB194951_Cameroon; A3_AM184125_Gabon; A4_AY934764_Gambia; A4_AM180623_Mali; A5_FJ692613_Haiti; A5_KP234053_Haiti; A6_GQ331047_Belgium; A6_GQ331048_Belgium; A7_FN545829_Cameroon; A7_FN545833_Cameroon; B1_AB642091_Japan; B1_D00329_Japan; B2_FJ899779_China; B2_JQ801485_Thailand; B3_GQ924617_Malaysia; B3_AP011085_Indonesia; B4_GQ924626_Malaysia; B4_AB115551_Cambodia; B5_GQ924640_Malaysia; B5_AB219427_Philippines; B6_JN792893_Canada; B6_DQ463787_Canada; B7_GQ358137_Indonesia; B7_AP011091_Indonesia; B8_GQ358147_Indonesia; B8_AP011093_Indonesia; B9_GQ358146_Indonesia; B9_GQ358149_Indonesia; C1_KP017266_India; C1_GQ377605_China; C2_GQ358158_Indonesia; C2_FJ899761_China; C3_DQ089801_Hong Kong; C3_EU939536_China; C4_HM011493_Malaysia; C4_Y18855_China; C5_EU410080_Philippines; C5_Y18856_China; C6_EU670263_Philippines; C6_AB493838_Indonesia; C7_GU721029_South Korea; C7_Y18858_China; C8_AP011104_Indonesia; C8_AP011105_Indonesia; C9_AP011108_Indonesia; C9_V00867_Japan; C10_AB540583_Indonesia; C10_KY670782_China; C11_AB554019_Indonesia; C11_AB554020_Indonesia; C12_AB554018_Indonesia; C12_AB560662_Indonesia; C13_AB644280_Indonesia; C13_AB644281_Indonesia; C14_AB644283_Indonesia; C14_AB644284_Indonesia; C15_AB644286_Indonesia; C15_D50489_Japan; C16_AB644287_Indonesia; C16_D23682_Japan; D1_GU456636_Iran; D1_JN642165_Lebanon; D2_GQ477452_Poland; D2_KF679995_India; D3_EU594434_Estonia; D3_X65257_Italy; D4_GQ922003_Canada; D4_KF192838_India; D5_GQ205377_India; D5_GQ205384_India; D6_KF170740_Sudan; D6_AB493845_Indonesia; D7_FJ904442_Tunisia; D7_FJ904425_Tunisia; D8_FN594770_Niger; D8_FN594771_Niger; D9_JN664942_India; D9_X59795_Italy; D10_KX357625_Ethiopia; D10_KX357629_Ethiopia; E_FN594748_Niger; E_HM363610_Nigeria; F1a_AY090459_Costa Rica; F1b_FJ709464_Chile; F2a_DQ899142_Venezuela; F2b_DQ899146_Venezuela; F3_AB036920_Venezuela; F3_AB036905_Venezuela; F4_AF223965_Argentina; F4_DQ823090_Argentina; G_GU563556_Belgium; G_KP274926_Belgium; H_AB516393_Mexico; H_AB516395_Mexico; I1_FJ023659_Laos; I1_FJ023660_Laos; I2_FJ023664_Laos; I2_FJ023672_Laos; J_AB486012_Japan.

HBV whole genome sequences and genotype of 53 isolates from current study:

C1_BD_HBV57; C3_BD_HBV19; C1_BD-HBV55; C3_BD_HBV53; C1_BD_HBV21; C1_BD_HBV09; C1_BD_HBV52; C1_BD_HBV13; C1_BD_HBV44; C1_BD_HBV31; C1_BD_HBV18; C1_BD_HBV02; C1_BD_HBV25; C1_BD_HBV14; C1_BD_HBV45; C1_BD_HBV26; C1_BD_HBV49; D2_BD_HBV24; D2_BD_HBV03; D2_BD_HBV48; D1_BD_HBV35; D1_BD_HBV28; D1_BD_HBV43; D2_BD_HBV27; D2_BD_HBV07; D2_BD_HBV38; D2_BD_HBV08; D2_BD_HBV34; D2_BD_HBV01; D2_BD_HBV37; D2_BD_HBV23; D2_BD_HBV47; D2_BD_HBV32; D2_BD_HBV15; D2_BD_HBV42; D5_BD_HBV10; A1_BD_HBV50; A1_BD_HBV05; A1_BD_HBV16; A1_BD_HBV04; A1_BD_HBV54; A1_BD_HBV29; A1_BD_HBV17; A1_BD_HBV46; A1_BD_HBV12; A2_BD_HBV30; C1_BD_HBV33; C3_BD_HBV20; C1_BD_HBV41; 1_BD_HBV11; C1_BD_HBV56; C1_BD_HBV39; C1_BD_HBV22. 1a

The accession number for the sequences in GenBank is MF925358 to MF925410.
