# Supplementary material for: Molecular characterization of hepatitis B virus in Bangladesh reveals a highly recombinant population
Source: PLoS One. 2017 Dec 7;12(12):e0188944. doi: 10.1371/journal.pone.0188944 (PMC5720799; doi:10.1371/journal.pone.0188944)
Supplement: S2 File — Potential NAr mutations including reported NAr mutations and novel NAr mutations identified in the present study among different genotypes are presented. (DOCX) [file pone.0188944.s002.docx]

S2 File

S 2File. Potential NAr mutations including reported NAr mutations and novel NAr mutations identified in the present study.

| Mutation category | Mutation types | Genotype A | Genotype C | Genotype D |
| --- | --- | --- | --- | --- |
|  |  | N = 10 | N = 24 | N = 19 |
| Primary drug resistance mutation | M204V |  | 1 |  |
| Secondary/compensatory mutation | V173L |  | 1 |  |
|  | L180M |  | 1 |  |
| Putative Nar mutation | S213T |  |  | 1 |
|  | Q215S |  |  | 3 |
|  | L217R | 1 | 1 |  |
|  | N238T |  | 1 |  |
|  | N139D |  | 1 |  |
| Pretreatment mutation | D134E |  | 2 | 1 |
|  | N139H |  |  | 1 |
| Novel mutation: New AA mutation type found in this study | D134N^c^ |  | 1 |  |
|  | A181C^a^ |  | 1 |  |
|  | Q215E^b^ | 1 |  |  |
|  | Q215G^b^ |  |  | 1 |
|  | N238H^b^ |  |  | 3 |
|  | A38E^c^ |  |  | 1 |
|  | D53K^b^ |  |  | 1 |
|  | D53N^b^ |  |  | 3 |
|  | I53V^b^ | 4 |  |  |
|  | H54Y^b^ |  |  | 3 |
|  | H126Y^b^ | 3 |  |  |
|  | R153Q^b^ |  | 2 |  |
| Total mutations |  | 9 | 12 | 18 |

^a^ primary drug resistance mutation; b putative Nar mutation; ^c^ pretreatment mutation
