# Supplementary material for: Molecular characterization of hepatitis B virus in Bangladesh reveals a highly recombinant population
Source: PLoS One. 2017 Dec 7;12(12):e0188944. doi: 10.1371/journal.pone.0188944 (PMC5720799; doi:10.1371/journal.pone.0188944)
Supplement: S1 Table — HBV isolates were considered recombinant if detected by 5 out of 6 program (RDP, BootScan, Max Chi, Chimaera, SisScan and Topol). Recombinant isolate, recombination group major and minor parents and identity, recombinant break points, size of the recombinant fragment and location of the recombination are presented. (DOCX) [file pone.0188944.s003.docx]

S1 Table

S1 Table. Recombination analysis of the 12 HBV isolates from the study using RDP4 v 4.55 program. HBV isolates were considered recombinant if detected by 5 out of 6 program (RDP, BootScan, Max Chi, Chimaera, SisScan and Topol). Recombinant isolate, major and minor parents and identity, recombinant break points, size of the recombinant fragment and location of the recombination are presented below.

|  |  |  |  |  |  |  |  |  |  | Identity^b^ with | | Recombination detected by | | | | | | |
| --- | --- | --- | --- | --- | --- | --- | --- | --- | --- | --- | --- | --- | --- | --- | --- | --- | --- | --- |
| No | Recom group | HBV isolate | Recom binant | Length | Start & end breakpoint in sequence | Start & end breakpoint in alignment | Location* | Major parent | Minor parent | Major parent | Minor parent | R | G | B | M | C | S | T |
| **1** | **G1** | **HBV25_C1** | **C/B** | **360** | **1397-1757** | **1419-1779** | **X gene** | **HBV20_C1** | **GQ924626_B4** | **98.2** | **98.9** | **N** | **Y** | **N** | **Y** | **Y** | **Y** | **Y** |
| **2** | **G1** | **HBV14_C1** | **C/B** | **438** | **1397-1835** | **1419-1857** | **X gene & BCP, PC start** | **HBV20_C1** | **GQ924626_B4** | **98.8** | **99.3** | **N** | **Y** | **N** | **Y** | **Y** | **Y** | **Y** |
| **3** | **G1** | **HBV45_C1** | **C/B** | **474** | **1379-1853** | **1401-1875** | **X gene & BCP, PC start** | **HBV20_C1** | **GQ924626_B4** | **97.6** | **96.8** | **N** | **Y** | **N** | **Y** | **Y** | **Y** | **Y** |
| **4** | **G1 ^β^** | **HBV26_C1** | **C/B** | **414** | **1396-1810** | **1418-1832** | **X gene & BCP, PC start** | **HBV20_C1** | **GQ924626_B4** | **96.9** | **99** | **N** | **Y** | **N** | **Y** | **Y** | **Y** | **Y** |
| **5** | **G2 ^β^** | **HBV43_D2** | **D/C** | **713** | **1250-1963** | **1272-2021** | **end P, X gene, BCP, PC start** | **HBV35_D1** | **HBV31_C1** | **98.3** | **100** | **N** | **Y** | **Y** | **Y** | **Y** | **Y** | **Y** |
| **6** | **G2** | **HBV32_D2** | **D/C** | **626** | **1296-1922** | **1319-1981** | **X gene & BCP, PC start** | **HBV35_D1** | **HBV31_C1** | **97.3** | **100** | **N** | **Y** | **Y** | **Y** | **Y** | **Y** | **Y** |
| **7** | **G2** | **HBV15_D2** | **D/C** | **673** | **1250-1923** | **1272-1981** | **end P, X gene & BCP, PC** | **HBV35_D1** | **HBV31_C1** | **98.3** | **100** | **N** | **Y** | **Y** | **Y** | **Y** | **Y** | **Y** |
| **8** | **G2** | **HBV42_D2** | **D/C** | **663** | **1250-1913** | **1272-1971** | **end P, X gene, BCP & PC** | **HBV35_D2** | **HBV31_C2** | **96.3** | **100** | **N** | **Y** | **Y** | **Y** | **Y** | **Y** | **Y** |
| **9** | **G3** | **HBV50_A1** | **A/C** | **547** | **1356-1903** | **1378-1928** | **end P, X gene, BCP & PC** | **HBV04_A1** | **HBV44_C1** | **99.4** | **100** | **N** | **Y** | **N** | **Y** | **Y** | **Y** | **Y** |
| **10** | **G3** | **HBV30_A2** | **A/C** | **547** | **1376-1923** | **1399-1982** | **X gene, BCP & PC start** | **HBV04_A1** | **HBV44_C1** | **95.2** | **99.6** | **N** | **Y** | **N** | **Y** | **Y** | **Y** | **Y** |
| **11** | **G4** | **HBV03_D2** | **D/B** | **1139** | **764-1903** | **786-1946** | **end S gene, end P gene, X gene, BCP, PC & C start** | **HBV08_D2** | **FJ899779_B2** | **99** | **96.4** | **N** | **Y** | **N** | **Y** | **Y** | **Y** | **Y** |
| **12** | **G4** | **HBV24_D2** | **D/B** | **1170** | **732-1902** | **754-1946** | **end S gene, end P gene, X gene, BCP, PC & C start** | **HBV08_D2** | **FJ899779_B2** | **98** | **98.6** | **N** | **Y** | **N** | **Y** | **Y** | **Y** | **Y** |

R; RDP: G: Gencove: B; Bootscan: M; Maxchi: C; Chimaera: S; Siscan: T; Topol: N; No: Y; Yes. ^b^ The identity was estimated using the consensus sequences of the non-recombinants of different genotypes, ^⁎^ we have listed the genes where the recombinant fragments are located, ^¥^ Length of the recombinant fragment. **^β^;** acute infection
