# Supplementary material for: Molecular characterization of hepatitis B virus in Bangladesh reveals a highly recombinant population
Source: PLoS One. 2017 Dec 7;12(12):e0188944. doi: 10.1371/journal.pone.0188944 (PMC5720799; doi:10.1371/journal.pone.0188944)
Supplement: S1 Fig — Schematic presentation of six HVB RT domain functional regions and (F, A, B, C, D, and E) five regions (F-A, A-B, B-C, C-D, and D-E) connecting the functional regions. Functional regions are presented as box and regions between functional regions as lines. The start and end amino acid positions of each functional region are presented at the top of each box. Novel mutations are presented as brown bars and previously reported mutations as blue bars. (DOCX) [file pone.0188944.s004.docx]

S1 Fig

S1 Fig: Distribution of all NAR mutations identified in RT region


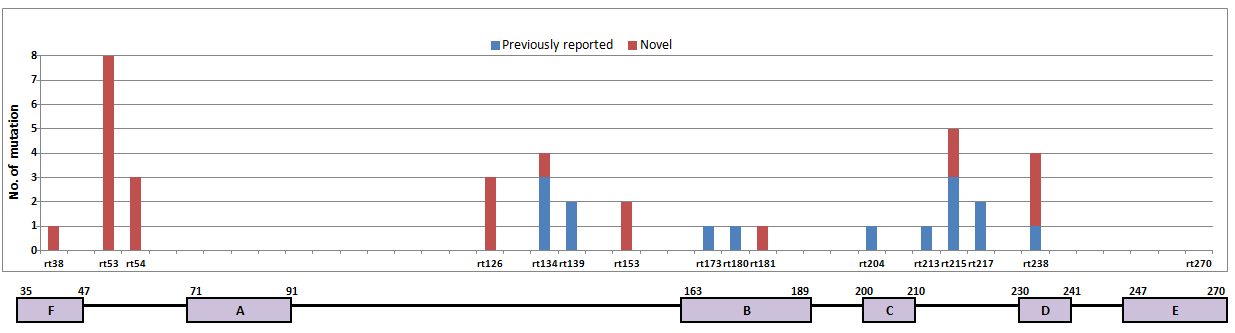


Schematic presentation of six HVB RT domain functional regions and (F, A, B, C, D, and E) five regions (F-A, A-B, B-C, C-D, and D-E) connecting the functional regions. Functional regions are presented as box and regions between functional regions as lines. The start and end amino acid positions of each functional region are presented at the top of each box. Novel mutations are presented as brown bars and previously reported mutations as blue bars.
